# "Do-It-Yourself" reliable pH-stat device by using open-source software, inexpensive hardware and available laboratory equipment

**DOI:** 10.1371/journal.pone.0193744

**Published:** 2018-03-06

**Authors:** Jovana Z. Milanovic, Predrag Milanovic, Rastislav Kragic, Mirjana Kostic

**Affiliations:** 1 Innovation Centre, Faculty of Technology and Metallurgy, University of Belgrade, Belgrade, Serbia; 2 Faculty of Technology and Metallurgy, University of Belgrade, Belgrade, Serbia; 3 Ministry of Mining and Energy, Belgrade, Serbia; The Ohio State University, UNITED STATES

## Abstract

In this paper, we present the construction of a reliable and inexpensive pH stat device, by using open-source “OpenPhControl” software, inexpensive hardware (a peristaltic and a syringe pump, Arduino, a step motor…), readily available laboratory devices: a pH meter, a computer, a webcam, and some 3D printed parts. We provide a methodology for the design, development and test results of each part of the device, as well as of the entire system. In addition to dosing reagents by means of a low-cost peristaltic pump, we also present carefully controlled dosing of reagents by an open-source syringe pump. The upgrading of the basic open-source syringe pump is given in terms of pump control and application of a larger syringe. In addition to the basic functions of pH stat, i.e. pH value measurement and maintenance, an improvement allowing the device to be used for potentiometric titration has been made as well. We have demonstrated the device’s utility when applied for cellulose fibers oxidation with 2,2,6,6-tetramethylpiperidine-1-oxyl radical, i.e. for TEMPO-mediated oxidation. In support of this, we present the results obtained for the oxidation kinetics, the consumption of added reagent and experimental repeatability. Considering that the open-source scientific tools are available to everyone, and that researchers can construct and adjust the device according to their needs, as well as, that the total cost of the open-source pH stat device, excluding the existing laboratory equipment (pH meter, computer and glossary) was less than 150 EUR, we believe that, at a small fraction of the cost of available commercial offers, our open-source pH stat can significantly improve experimental work where the use of pH stat is necessary.

## Introduction

Free and open-source software (FOSS) is defined as computer software that is available in source code (open-source) form and that can be used, studied, copied, modified, and redistributed without restriction, or with restrictions that only ensure that further recipients have the same rights under which it was obtained [1]. Nowadays, FOSS is becoming the norm in software development, in contrast to the closed box and secretive standard commercial approach to development [2–4], with the wide areas of application, such as: science and education [5, 6], enterprises [2] and medicine [7], engineering and nanotechnology [8–10], ecology [11], etc. FOSS represents a transparent system that enables creating both software and hardware. The development of open-source hardware has the great potential to significantly reduce the cost of performing experimental science and make high-quality scientific tools available to everyone, from the most prestigious laboratories to the laboratories in the resource-poor developing world [6, 12].

According to the literature, there is a great number of various open-source “do- it- yourself” devices, such as: a potentiostat [13–15], syringe pump [16], a colorimeter [17], as well as additional laboratory equipment [4, 18, 19], which researchers can construct and adjust to their needs at a small fraction of the cost of available commercial offers. However, to our knowledge, the open-source pH device for maintaining pH values has been constructed in a considerably smaller scope [20].

Considering that the average cost of commercially available pH stat unit and software is more than 9,000 EUR, while the complete equipment, necessary for using pH-stat, is about 20,000 EUR, we think that the cost represents a limiting factor for pH stat adoption in the resource-poor developing world. In order to improve the experiments in which the use of pH stat is necessary, the experiments in laboratories where the resources are limited, as well as, for the needs of our laboratory, we have designed the open-source “OpenPhControl” software and inexpensive pH stat device by using cheap laboratory equipment. The idea for designing the open-source pH stat arose due to a need for maintaining a constant pH value during the performance of the TEMPO-mediated oxidation of cellulose in our laboratory.

With this motivation in mind, in this paper, we present the construction of a reliable pH stat device by using inexpensive hardware, readily available laboratory devices and some 3D printed parts, along with the created open-source “OpenPhControl” software. The methodology for the design, development and testing of each part of the system, as well as of the entire system, has been provided.

A reagent dosing system is presented in two ways: by using a low-cost peristaltic pump and carefully controlled dosing of reagents by using an open-source syringe pump, manufactured according to the literature [16]. Additionally, the upgrading of the basic open-source syringe pump is given in terms of pump control and application of larger syringe. Apart from the basic functions of pH stat, i.e. pH value measurement and maintenance, an improvement has been made, allowing the device to be used for potentiometric titration. Finally, in support of usability of our open-source pH stat, we have demonstrated its utility in applications for TEMPO-mediated oxidation of cellulose fibers. Therefore, a brief description of the TEMPO-mediated oxidation, its aim and application is presented below.

TEMPO-mediated oxidation is the established name for the catalytic and selective oxidation of primary hydroxyl groups of carbohydrates, using stable 2,2,6,6-tetramethylpiperidine-1-oxyl radical (TEMPO), NaBr and NaClO, by which aldehyde and carboxyl functional groups can be introduced into solid native polysaccharides [21–25]. A powerful TEMPO-mediated oxidation nowadays is applied by many researchers, [26–30], as the first step on the way to obtain cellulose nanofibrils (CNF) [26]. In the field of nanofibrils, cellulose, which is the most abundant renewable biopolymer, has attracted a great attention as one of the promising polysaccharides for accomplishing highly engineered nanofibrils and nanoparticles. Due to their excellent properties, including biodegradation, biocompatibility, the large specific surface area, unique optical properties, etc., cellulose nanofibrils and nanoparticles can be applied in many areas [31].

According to the procedure [22, 23, 32], during the TEMPO-mediated oxidation, it is very important to maintain the pH value between 10–11 (in our experiments it was 10.55), since undesirable side effects occur at lower pH, i.e. hypochlorite becomes an overly aggressive and non-selective oxidant, and also TEMPO reactivity is decreased [21, 22]. In the absence of pH stat, during the oxidative process, pH values must be maintained manually by adding sodium hydroxide solution from the burette, according to the value displayed on the pH meter. Since the experiments lasted for several hours, the permanent presence of the operator was necessary all the time. In such a long period, the subjectivity of the operator can come to the fore, so it is possible that repeatability of experiments is not satisfactory. In order to avoid the operator’s constant attention over the entire experiment, as well as the operator’s subjective error that can be made during the pH value maintaining in the oxidative process, now we use the open-source pH-stat.

In this paper we also present the results obtained for the oxidation kinetics, the consumption of added sodium hydroxide and experimental repeatability, for the TEMPO-mediated oxidations of cellulose carried out by using open-source pH stat.

## Material and methods

### Design

The design goals were to create a reliable pH stat device which will be able to maintain the pH of a solution by using open-source software “OpenPhControl”, inexpensive hardware (a peristaltic pump, Arduino, a step motor…), some 3D printed parts and available laboratory equipment (a pH meter, a computer, a webcam…). Considering that the construction of a pH meter is challenging and that it is standard equipment of every research laboratory, the decision was made that the measured pH value should be “read” by personal computer using a webcam. In this way, the reliable measuring of pH is achieved by tested equipment, with all protocols of maintenance and calibration. Furthermore, the open-source software “OpenPhControl” was designed to use pump modules, which were controlled by Arduino microcontrollers [33] with a USB connection. The two pump modules were designed (peristaltic and syringe pump modules) by defining its hardware and specialized firmware that enables its usage. Finally, considerations were made for ease of assembly and reduced cost without compromising performance.

**Fig 1:**
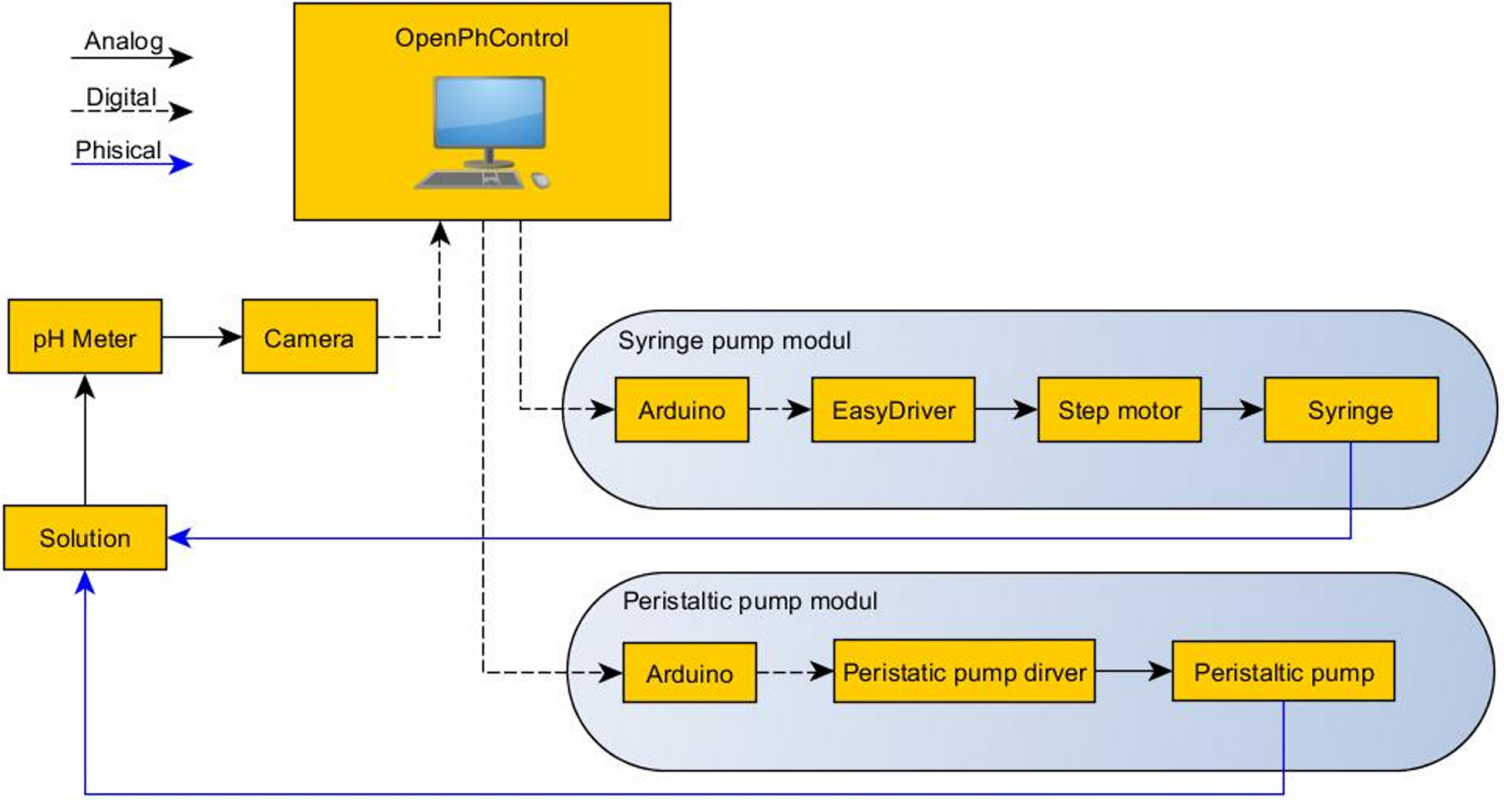
Schematic overview of the key components of the system, including the computer (PC) with “OpenPhControl”.

The major components of “OpenPhControl” system are shown in Fig 1 and an examination of each component follows.

#### Open-source “OpenPhControl” software

Open-source “OpenPhControl” software is a key part of the whole system. It is the software written in Java and consists of three parts. The first part processes a video signal and optically recognizes characters (digits) from a display of a pH meter. The second part represents the logic of the system—makes decisions about dosing of reagents while maintaining a pH value. The third part communicates with the pump modules, while the Graphical User Interface (GUI) connects everything in the working system.

The GUI is created by using a GUI widget toolkit for Java—SWING. It is a part of Oracle's Java Foundation Classes (JFC)–an Application Program Interface (API) for providing a platform-independent Graphical User Interface for Java programs. A custom-written Graphical User Interface (GUI) allows control of all important process parameters, manipulation of pump modules, as well as, manipulation of recorded data (graphs display, continuation of experiments if needed…). With the aim to present the current capabilities, a few screenshots are presented in Support of information Figures A-J in S1 File. The control over other process parameters can be added if required.

The commands to control each peripheral module are created in a standard manner, making the standard way of controlling the modules—the end user is able to create new hardware modules that would work without modification of “OpenPhControl”. The communication between the PC and the Arduino is established via Universal Serial Bus (USB) through a platform-independent serial port access library for Java jSerialComm [34] which is licensed under the GNU General Public License v3.0. Both libraries are licensed under the GNU General Public License.

JFreeChart was used for creating graphs [35]. JFreeChart is an open-source framework for the programming language Java, which allows the creation of a wide variety of both interactive and non-interactive charts. It is distributed under the terms of the GNU Lesser General Public License (LGPL), which permits use in proprietary applications.

All recorded data (read pH and added volume) are stored in csv ASCII files that are suitable for use in other mathematical and statistical software.

We used Debian [36] as the Operating System (OS) because it is a stable and well-supported GNU-Linux distribution, suitable for work on old computers. While working on “OpenPhControl”, it was taken into consideration that only platform-independent frameworks and modules could be used, so the whole system will work on other OS which supports Java. It was successfully tested on Windows 7 and Windows 10.

### pH measurement

The basic function of pH meter devices, i.e. the measurement of a pH value of solution, is typically achieved by using specialized electrodes in which the difference in electrical potential between a pH electrode and a reference electrode is transformed in a corresponding pH value. Regardless of the operation method, the one thing that all pH meters have in common is that they have a display for displaying measured values.

This work is based on the assumption that every laboratory has a pH meter that is capable of stable and reliable pH measurement. In this paper, a pH meter MA 5740 (Iskra, Slovenia) was used for the testing purpose. The mentioned pH meter has a red seven segment [37] display with precision of ±0.001 pH.

### Video and image acquisition

Webcam Capture API library allows the usage of a build-in or external webcam directly from Java. It is designed to abstract commonly used camera features and support multiple capturing frameworks on multiple platforms (Windows, Linux, Mac OS, etc.) and various architectures (32-bit, 64-bit, ARM) with no additional software required. It offers simple, thread-safe and non-blocking API for getting images from build-in or USB-connected PC webcams, as well from IP/network cameras [38]. It is published under permissive free software license—the MIT License (MIT).

Overall image processing was obtained on the RGB video frames (640 x 480 pixels) directly from the memory.

#### Digit recognition

The CANYON camera (CHR-WCAM43G, f4.8mm) was selected to display the pH meter, and measured pH value was recorded and processed with “OpenPhControl” software. For the basic image/video manipulation, pure Java cross-platform image processing framework that provides features for image and video frame processing, multi-threading image processing, GUI integration—Marvin [39], was used. Marvin is released under the GNU Lesser General Public License (LGPL).

The code for Optical Character Recognition (OCR) that recognizes digits on a 7 segment display was developed. The used pH meter has a display with red digits, so a red layer of recorded frame was used. With adjustable threshold of pixel intensity and scheme of segments it was determined which segments were “on” and according to it the digit was recognized.

### Modular pump modules

Every module is connected to a computer with one USB cable. The number of connected modules is limited by the number of free USB ports on a host computer. The “OpenPhControl” software automatically reads from the pump module all the data it needs to carry out the work properly, i.e. a type of a pump, a type of a reagent, calibration parameters.

We created standard commands for the communication between “OpenPhControl” and pump modules. Therefore, creating new types of a pump module does not affect the existing “OpenPhControl” software.

Every module, while being connected to “OpenPhControl”, identifies itself and exchanges all data necessary for the proper functioning. It makes it possible for the operator to change a pump module easily or to work simultaneously with the numerous types of pump modules with different characteristics. For example, during titration, more than one type of pump modules could be connected to “OpenPhControl” (i.e. two syringe pumps with 20 ml syringe, a syringe pump with 50 ml syringe and peristaltic pump) and the operator can, through GUI, easily switch the pump module which is used for dosing.

#### Arduino

To accomplish controlling of the pumps, we used an ATmega328-based Arduino Nano [33]. Arduino microcontrollers are a family of open-source, low-cost integrated circuits that contain a core processor, memory and analog and digital I/O (input/output) peripherals. The board was chosen due to its low cost and easy availability (including several ultra-low-cost clones at €2 to €3), its openness (it is open hardware), very well-documented environment (hardware specifications, function descriptions). The board is programmed in C++ together with the Arduino integrated development environment (IDE). Once the board is loaded with the correct code, which can be done on any computer, the Arduino IDE is not used any more, as all live communication goes via the Universal Serial Bus (USB) port directly from “OpenPhControl”. Arduino is a basic component of pump module and is responsible for controlling peripheral device (peristaltic and syringe pumps)–it takes the standardized commands by USB interface and executes it. The code can be used for most of the other boards of the Arduino family without any modification. The code is available on “OpenPhControl” repository https://gitlab.com/mpele/OpenPhControl.

#### Syringe pump module

**Fig 2:**
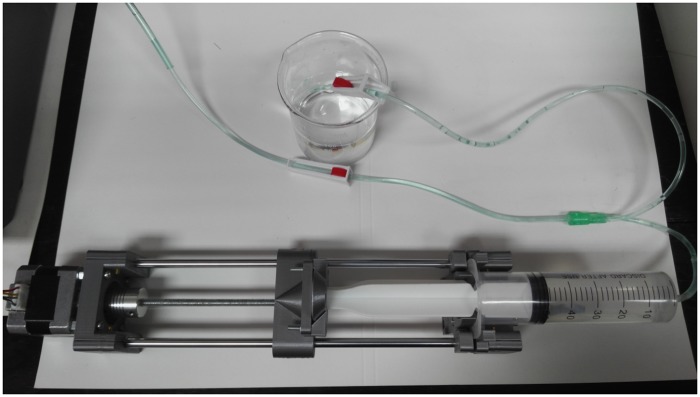
The upgraded open source syringe pump [16] with a 50 ml syringe and tubes with valves and tubes from infusion set.

**Fig 3:**
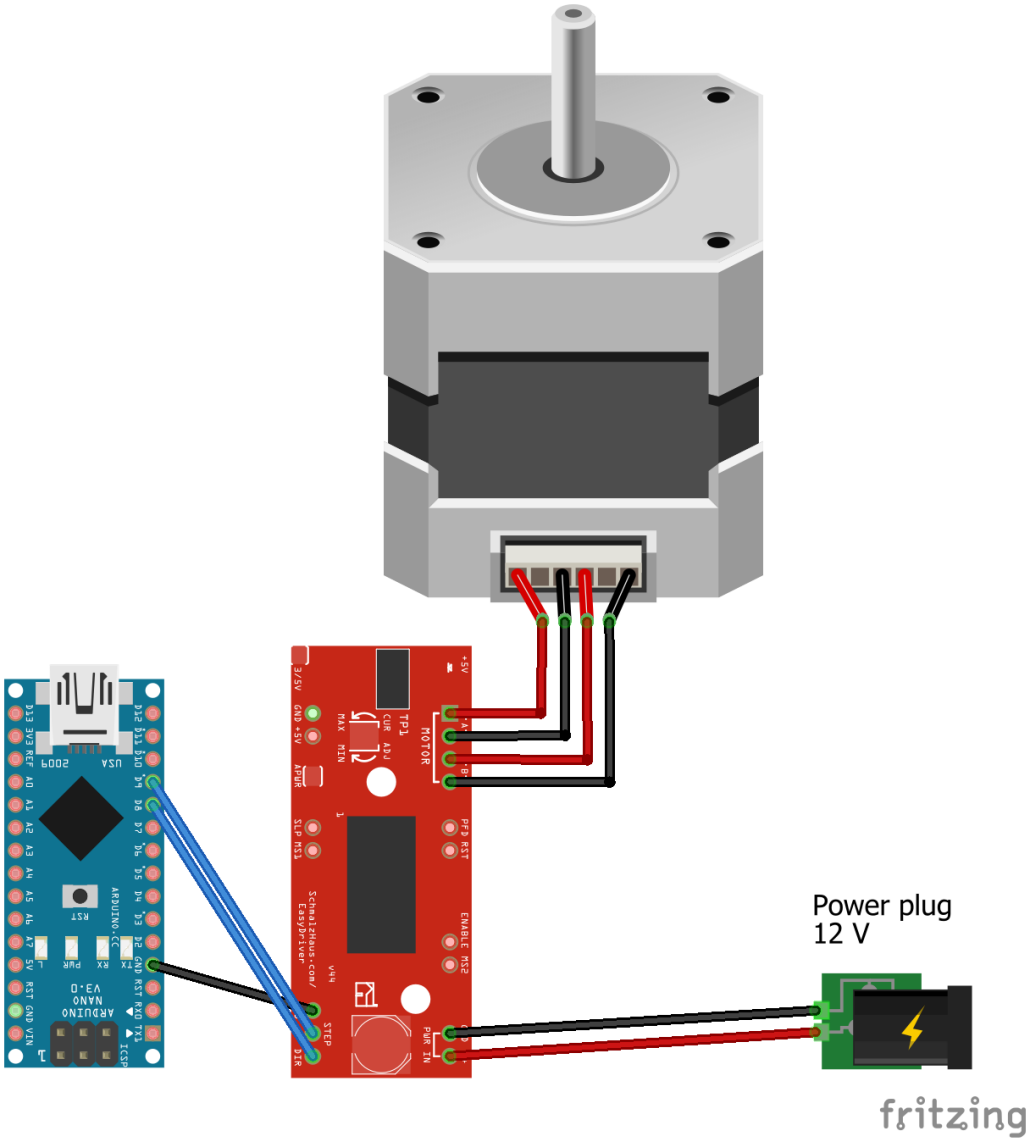
Wiring of syringe pump module.

The syringe pump module consisted of the syringe pump and Arduino. The syringe pump was made with the step motor NEMA 17 in accordance with the instructions published in the article [16]. The pump presented in the mentioned article uses a 20 ml syringe, which was not enough for our purpose, so we made some modifications for using a 50/60 ml syringe (Fig 2).. The additional 3D modeling was performed in OpenSCAD [40]. All parts were printed in polylactic acid (PLA) on a 3D printer MakerBot Replicator 2. The pump driving was done by Arduino Nano board which, through EasyDriver 4.4, controlled the step motor of the syringe pump (Fig 3). The commands to Syringe pump module were controlled by “OpenPhControl” by sending the commands through the USB to Arduino board.

The syringe pump module is identified automatically when it is connected to the “OpenPhControl”. The basic data about the module (name, description and reagents) and calibration data are stored in the EEPROM of Arduino which makes the Syringe pump module transferable—it does not need to be recalibrated each time when different module is used or when the module is used on different PC.

By controlling the number of steps of motor, the dosed volume is controlled as well, and by speed of motor, the speed of dosing reagents is controlled.

#### Peristaltic pump module

As one of the main goals was to make the system cheaper, we used a peristaltic pump for laboratory and aquarium use. The pump can be sourced from overseas for less than 10 EUR including shipping. The pump uses a permanent-magnet AC motor with maximum 5000 RPM and with the tube size of 2.5x4.7mm. According to specifications, it should provide the flow rate in the range 20–60 ml/min.

The control of the peristaltic pump was achieved by Arduino Nano, by using a simple printed board presented in Supporting Information S1 Fig. Added volume is controlled by a pump operating time, with an accuracy of 1 ms. Since the pump speed is a function of voltage, the working voltage is set at 12 V by PC power supply or at 9V by Buck Step-down module (based on LM2596 chip) from a cheap power adapter.

The Peristaltic pump module, just as the Syringe pump module, has the same capabilities for automatic identification when it is connected to “OpenPhControl”. The commands for the pump control are the same as for the Syringe pump module, with the exception of defining the dosing speed during work. The assembly of the Peristaltic pump module takes about 30 min for a person with no previous soldering experience, provided that all individual components are in place.

### Control of dosing

The addition of reagents in a solution could be controlled manually or automatically. In manual control, the addition of reagents could be controlled by pressing the button on the keyboard. As long as the button is pressed, the pump is working and dosing of a reagent is continuous. For rough control, the automatic adaptive control mechanism is made. In this working mode, if the pH is below the defined value and if enough time has passed since the previous dosing, the system will dose the defined volume.

### Preparation of TEMPO-oxidized cellulose fibers

TEMPO/NaBr/NaClO-oxidation was carried out according to previously published methodology [22, 25]. In brief, 10g of cellulose fibers (cotton linters obtained from “Viskoza a.d.” Loznica, Serbia, with 0.0063 mmol CHO groups/g fibers and 0.0401 mmol COOH groups/g fibers) were suspended in water (750 ml) containing 0.050g TEMPO (C_9_H_18_NO, 98%, ″Aldrich″, Germany) and 0.50 g sodium bromide (NaBr, M = 102.89 g/mol, ″Acros″, Organics, USA). In our experiment, the TEMPO-mediated oxidation was carried out on the cellulose slurry in a total volume of 2.5 dm^3^. Subsequently, the designed amount of 12% available chlorine NaClO solution (NaOCl, M = 74.44 g/mol, ″Acros″, Organics, USA), corresponding to 12 mmol/g cellulose, whose pH had previously been adjusted to 10.55 [41], was added to the mixture under continuous stirring. The reaction rate was then monitored by the consumption of a solution of 0.5 M NaOH (NaOH, M = 40.00 g/mol, ″Lachema″, Czech Republic), while maintaining the pH of the slurry at 10.55 at room temperature for 3 h and using open-source pH-stat. After stirring within the designated time, the oxidation was quenched by adding ethanol (ca. 5 ml, C_2_H_5_OH, 95%, M = 46.07 g/mol, ″Zorka Pharma″ a. d. Sabac, Serbia). The oxidized cellulose was washed thoroughly with water and then ethanol on filter paper was placed in the Büchner funnel. The resultants were stored as a never-dried state at 5°C. The carboxyl and carbonyl content of each sample was determined according to the method described in literature [32]. All used chemicals were of analytical grade and used without further purification.

## Results and discussion

The “OpenPhControl” source code is available on the link. The pumps modules were built as described in the list of parts and assembly instructions in the literature [16] and Supporting Information S1 Fig. They were operated by running the “OpenPhControl” software via the USB connector.

### Calibration of camera

**Fig 4:**
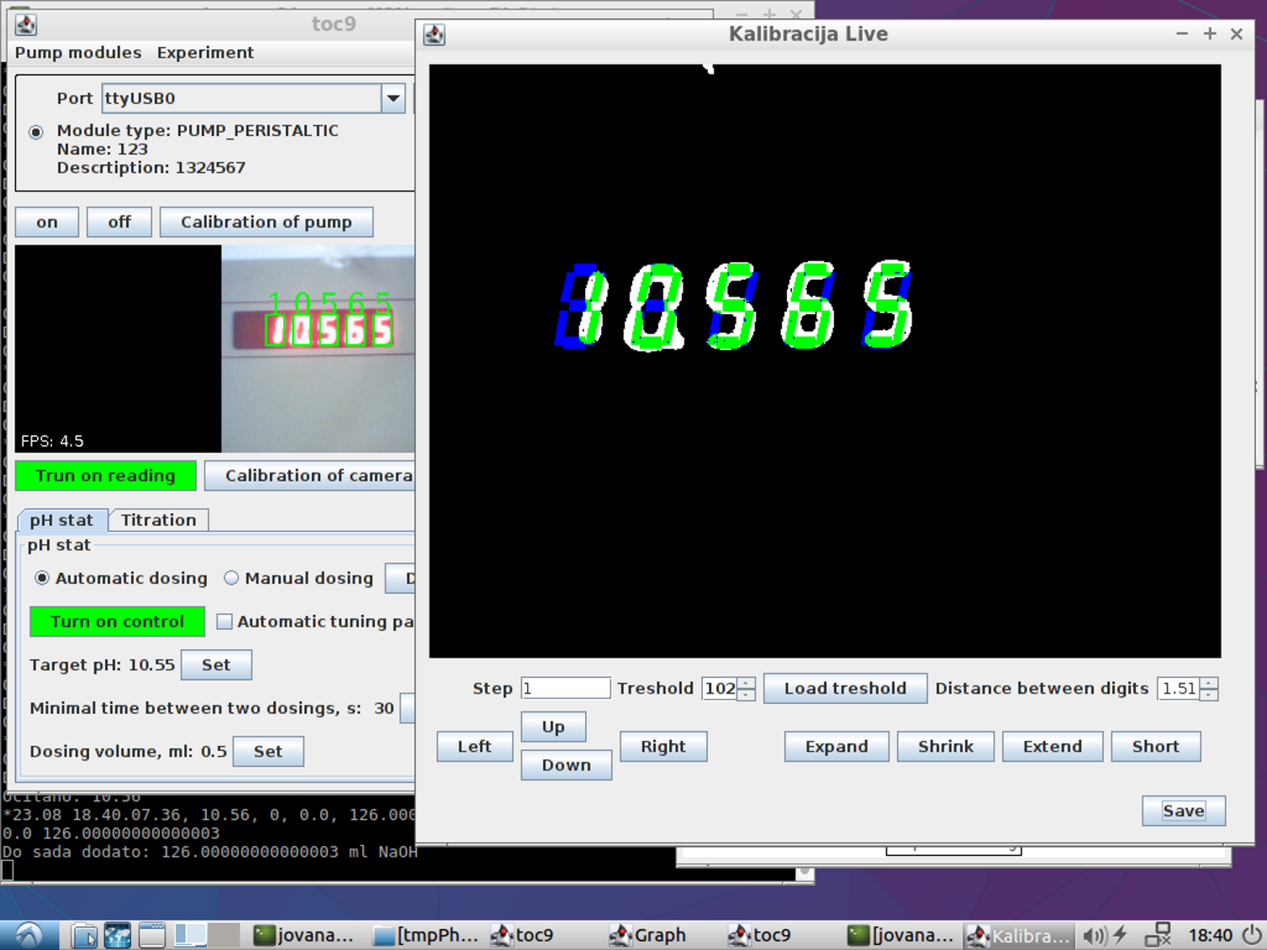
The print screen of calibration of digits reading.

Under GUI, it is possible to calibrate readings, i.e. to define locations of segments on the current video frame and threshold value for the determination of pixels from segments that are on. The first step is setting the threshold value so that the digits are clearly visible. For calibration the red layer of recorded frame is shown in separate window and threshold should be chosen in range from 0 to 255 to displayed figures be clear. After defining the threshold, the exact location of segments should be defined. It is accomplished by defining a location of one digit, its height and width, as well as, distance between digits. The example of the print screen of the calibration of digits reading is given in Fig 4.

If the reading has been adjusted correctly, the readings are stable and reliable. The bad reading may occur due to a slow transition of values on the pH meter display, but those readings are ignored. Frequency of reading was, depending on light, 6–15 frame per second, so the next frame with the clear image was in about 0.1 seconds and therefore, some incorrect bad reading had no effect on the whole process.

During the experiments, which lasted for a few hours, in some cases it was needed to correct the threshold due to a change of light (its intensity or switching between natural and artificial light).

According to the characteristics of our camera (pixels resolution 640x480), although the image quality with this resolution is obsolete today, we have concluded that this pixel resolution is quite sufficient for reading the displayed 7 segment digits.

### Calibration of pumps

#### Peristaltic pump module

The calibration of the peristaltic pump was done through “OpenPhControl” GUI interface. Calibration was done by adding a volume of water equivalent to the engine running for 500ms. The number of repeated addition of water is pre-defined (e.g., 50 times). The measured volume is used to calculate the coefficient ml/ms, which is saved on Arduino EEPROM memory.

**Fig 5:**
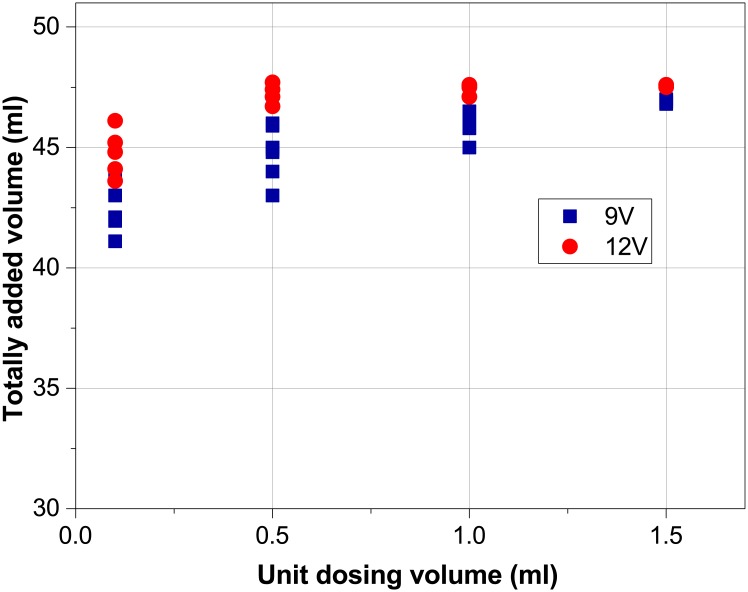
Precision testing of peristaltic pump powered at 9V and 12V.

Fig 5 shows the results of testing the peristaltic pump precision. The working voltage was set at 9V and 12V. The testing was done using the “OpenPhControl” GUI interface by defining the total volume to add 45 ml by dosing 0.1, 0.5, 1.0 and 1.5 ml with 1s pause between two dosings. As it can be seen from Fig 5, for 9V tests, the dissipation of total added volume is significant at small dosing unit volumes, while at dosing by 1.5 ml the repeatability is in 1 ml.

The tests on 12V showed better results, but still the added value had the dissipation with unit volumes smaller than 0.5 ml.

By increasing the dosing unit volume, the added volume is increased by 3–5 ml and the dissipation is reduced when 9V power supply is used, while the dissipation with 12V power supply is smaller.

The presented effect could be explained by the type of the motor used in the pump. The used peristaltic pump uses permanent-magnet AC motors, which have the unstable start. The first few ms, until the motor gets into the stable operation mode, are unpredictable, so when the ratio between the start period and stabile period is smaller the dosing is more precise. Also the quality of power supply could influence the dissipation of added volume, so we used 350 W power supply from an old PC, which provided constant 11.87 V. The maximum dosing rate with the mentioned power supply is 49 ml/min.

The usage of the peristaltic pump is acceptable for maintaining pH, but the result showing the added volume of reagents is not reliable if small doses are used (0.1–1 ml for 9V and 0.1 for 12 V).

The used Peristaltic pump module is suitable to be used in processes where the precision of added volume is not crucial, and when expected volumes, which should be added, are greater than the volume of available, more precise rechargeable pumps. The cost of this module (~15 EUR) makes it appropriate and justified for use in laboratories with the limited resources.

Better precision of a peristaltic pump should be achieved with pumps with step motors, but those pumps are more expensive (~40EUR) compared to peristaltic pumps with permanent magnets and require different type of control (similar to a syringe pump).

#### Syringe pump module

The calibration of syringe pumps was done with 20 ml and 50 ml syringe and motor NEMA 17 version. The calibration was done by measuring the ejected mass of distilled water [16]. The accuracy was +/−5% for the NEMA17, measured in 1 ml increments. The coefficient of variation when delivering approximately 10 ml of distilled water was about 1%. Considering that the step motor NEMA 17 has 200 step/revolution, with pitch of 0.8 mm [42] pushing the syringe in resolution of 0.004 mm/step, the minimal theoretical volume resolution is 0.0013 ml/step with 20 ml syringe or 0.0026 ml/step with 50 ml syringe. The mentioned minimal volume resolution is not relevant because the formation of a drop (surface tension) will define the practical volume resolution.

A maximum delivery rate of 0.75 ml/s, and 1.5 ml/s was obtained, respectively. The measurement method was limited to the volume of a single drop (e.g. 20 μl), which is in agreement with literature data [16].

### Open-source pH stat application in TEMPO-mediated oxidation

**Fig 6:**
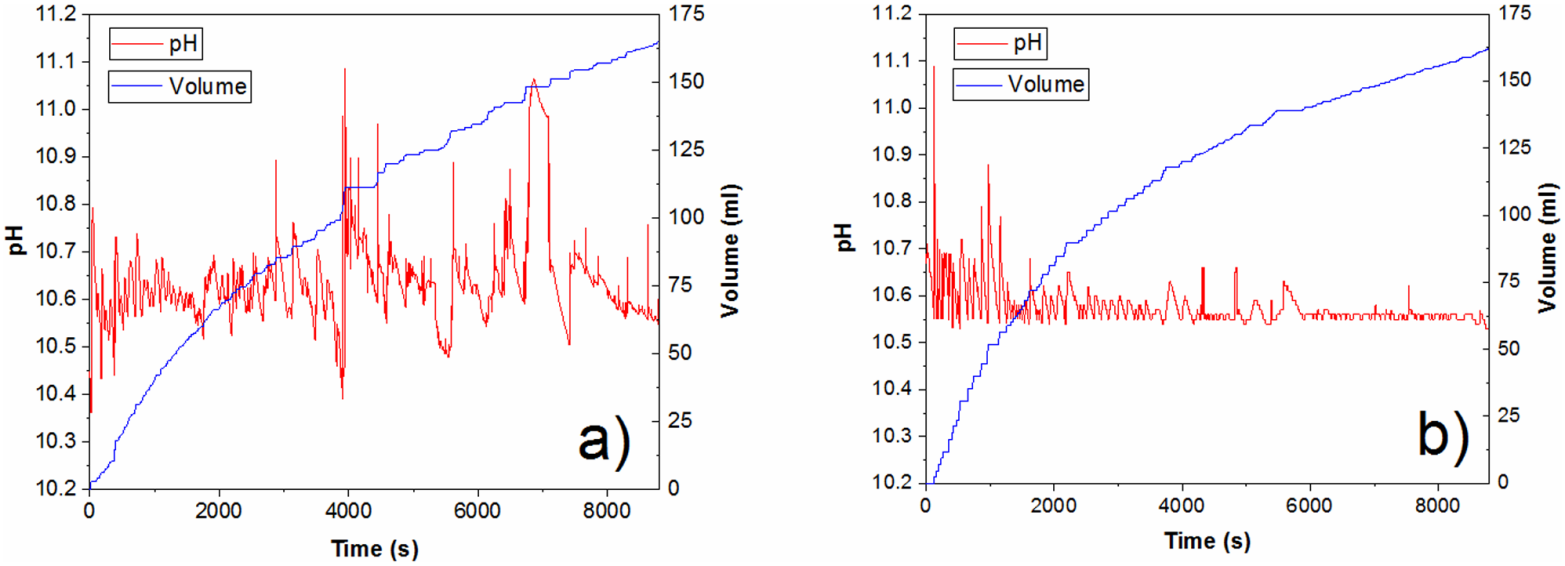
TEMPO-mediated oxidation of cellulose performed by “OpenPhControl” a) manually and b) automatically.

An example of the open-source pH stat application is shown for the TEMPO-mediated oxidation of cellulose. As we mentioned previously, during the TEMPO-mediated oxidation, it is very important to maintain the pH value between 10 and 11 (in our experiments it was 10.55) by adding 0.5M NaOH, since at a lower pH value undesirable side effects occurred [22, 23, 32]. Fig 6a presents the oxidation process through manual control of the “OpenPhControl” by pressing the keyboard for dosing NaOH solution, while Fig 6b shows the oxidation process driven by the “OpenPhControl”. The obtained results show that both ways of NaOH adding allow the maintenance of the required pH values, while automatic control provides more precise pH value control during whole oxidation, and completely excludes the subjective error of the operator. However, oxidation by manual addition was very important and necessary in order to automate the process.

**Fig 7:**
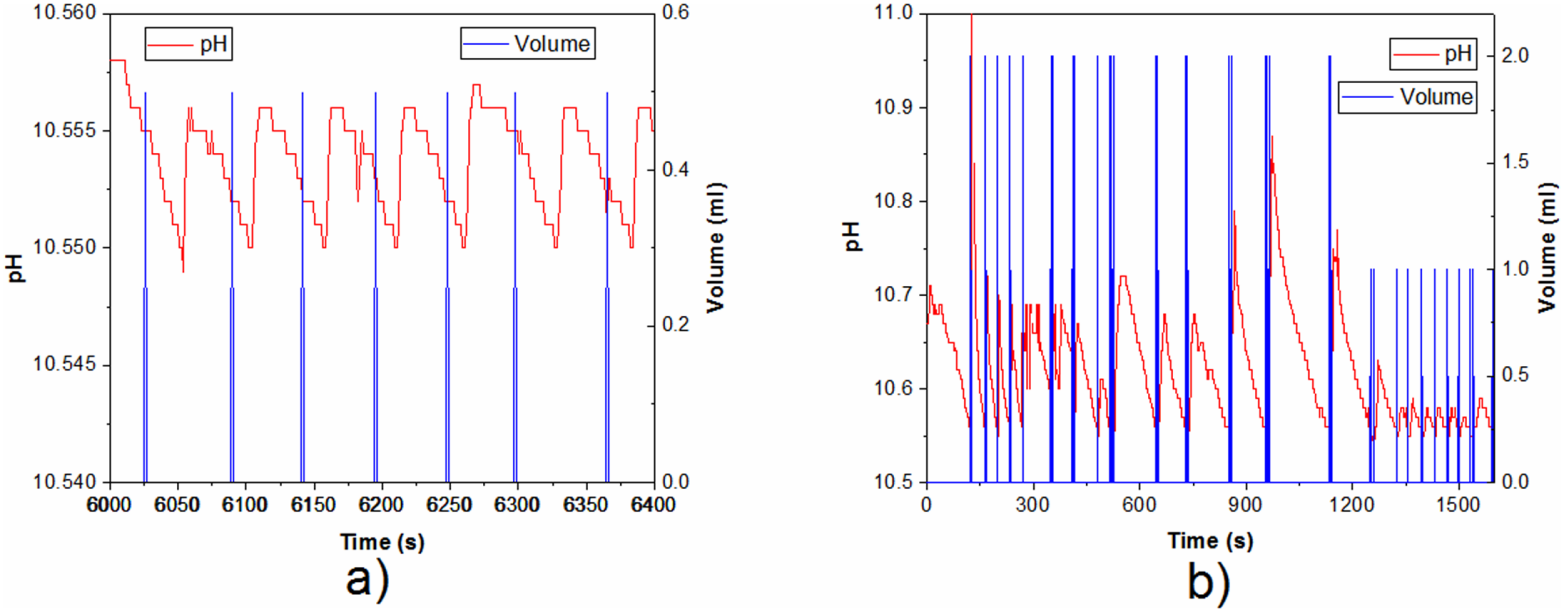
Zoomed graphs which demonstrate the change of pH (red lines) with addition of NaOH (blue lines) during TEMPO-mediated oxidation of cellulose: a) from 0 to 1500 s, and b) from 6000 to 6400 s.

According to the slope of added NaOH during the first phase of manually controlled test reaction, speed of dosing was estimated necessary (unit dosing volume and minimal time between two dosings) to provide necessary NaOH for expected pH dropdown. The details that show the progress of the TEMPO-mediated oxidation, i.e. the amount of added NaOH and the maintenance of pH value at the beginning of the oxidation, in the period from 0 to 1500 s, are shown in Fig 7a. As it can be seen, at the beginning of the oxidation, there was a need for more frequent and larger quantities of added NaOH. Therefore, the initial unit volume was set to 2 ml and minimal time between two dosings to 5 s. The predefined unit volume is decreased and minimal time between two dosings is increased by “OpenPhControl” if the system has more stable and predictable response. For example, during the oxidation, in the period from 6000 to 6400s, (Fig 7b), the unit volume was set to 0.5 ml.

This way of adjustment enabled the constant maintenance of the pH above 10.55 throughout the whole reaction, thus fulfilling the basic condition for the proper performance of the TEMPO-mediated oxidation. After the first phase of the oxidation, in our case, the pH value was constantly maintained in the range from 10.55 to 10.65, until the end of the reaction.

**Fig 8:**
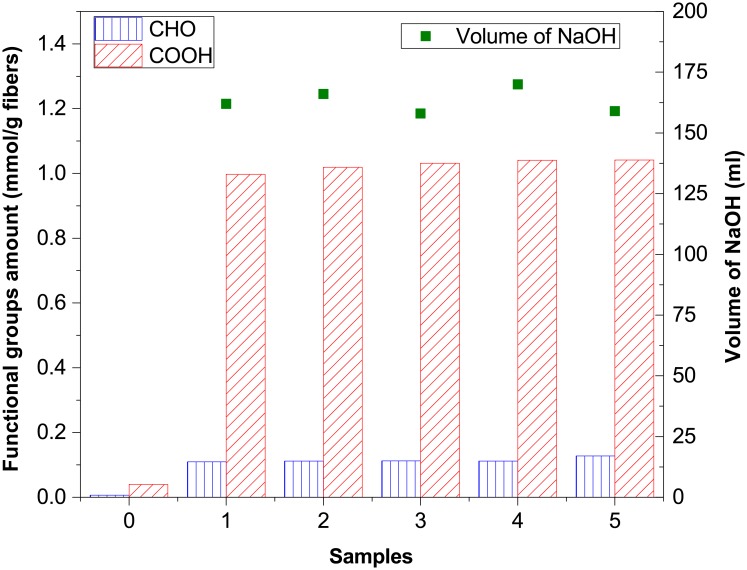
Functional groups amount in unmodified (0) and in the TEMPO-mediated oxidized cellulose fibers (samples 1–5), and the amount of consumed NaOH solution for maintaining pH during oxidation.

In addition, "OpenPhControl" also records data of the amount of added NaOH. Time frame for recording and processing data is 1 s. As a measure of the TEMPO-mediated oxidation repeatability, the consumption of 0.5M NaOH solution and the content of the introduced CHO and COOH functional groups were used (Fig 8). The amount of consumed 0.5M NaOH, for five parallel oxidations, was 165 ml ± 7.5 ml, and it was added by the peristaltic pump. The results obtained for the amount of introduced functional groups in the TEMPO-mediated oxidized cellulose samples, showed a small increase in CHO groups (up to 0.1278 mmol/g fibers), and considerable increase in COOH groups content (up to 1.0414 mmol/g fibers) in modified samples, compared to unmodified samples (0.0063 mmol/g fibers for CHO groups and 0.0401 mmol/g fibers for COOH groups). The obtained results show a very good repeatability of experiments which, with high content of introduced COOH groups, enable the successful preparation of cellulosic samples for the next steps on the pathway for the production of cellulose nanofibrils, which was the goal.

According to what is presented, by setting starting and working experimental conditions, the “OpenPhControl” could be used for acquisition of data for determination of the kinetics of acid-base or redox reactions.

### Titration

The “OpenPhControl” can perform titrations. Titrations are carried out by adding predefined volumes and by recording pH. Before next adding, the pH value should not be changed for a certain number of seconds. During titration, the predefined volumes could be changed by using “OpenPhControl” GUI.

**Fig 9:**
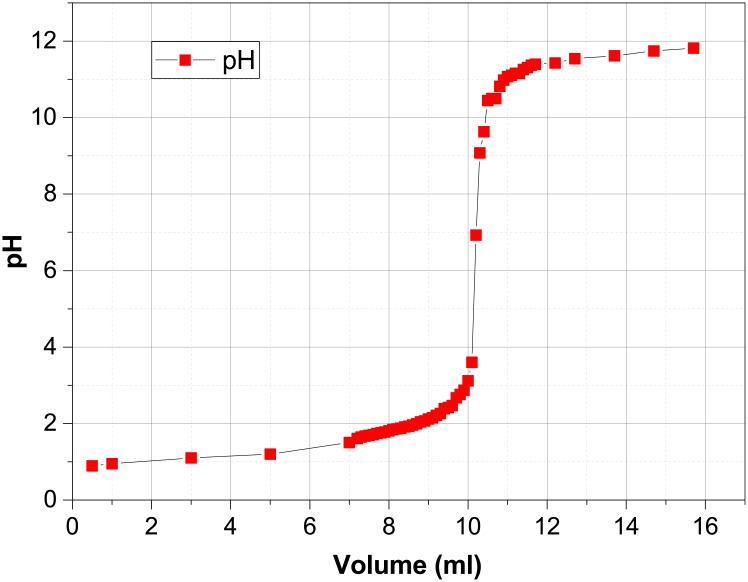
Titration curve recorded with “OpenPhControl”.

The titration of 10 ml 0.1 M HCl was carried out with 0.1 M NaOH by “OpenPhControl”, using a syringe pump module with a 20 ml syringe. The recoded titration curve is presented in Fig 9.

### Extensibility

A great advantage of open-source instruments is complete access to both the electronic hardware and the software used to operate them, giving exceptional flexibility in extending the instruments to new uses [6]. “OpenPhControl”—the full hardware schematics and source code for the computer software and modules firmware are freely available for modification. For example, “OpenPhControl” could be easily adapted to control pH as a function of time, or a new module type with pumps, stepper motors, piston pumps etc., could be added.

As we mentioned previously, in this work we used the pH meter that has a red seven segment display. Those displays are obsolete today and, on newer devices, they are replaced with different display technologies which work with pixels and have the resolution defined with physical number of columns and rows of pixels creating the display. Therefore, the suggested OCR system needs to be improved to work with modern displays.

We adapted “OpenPhControl” to read the display Titrino plus 848 (Metrohm, Switzerland). The Tesseract was used with Tess4J—Java JNA wrapper [43] for Tesseract OCR API. Tesseract is an optical character recognition engine for various operating systems [44]. The both programs are free software, released under the Apache License, Version 2.0.

The recorded images have to be prepared before processing with Tesseract (crop, converting to grayscale, etc.) and we succeed in obtaining correct readings by using the package with English training data. The mentioned display has resolution of 230x100 pixels with text height of 8 pixels, so we had to intensively use filters (erode, dilate, blur) to get correct readings. The general conclusion was that it is better to provide training to Tesseract for a display in use.

It has to be mentioned that the used webcam CANYON CHR-WCAM43G with the resolution of (640x480 px), which was used for reading a 7 segment display, was not sufficient for reading Titrino 848 display.

Considering the fact that the installation of Tess4J with Tesseract is slightly different on different operating systems (Linux, Windows and its architectures x86, x64) and that unique image preparation needed for each display could not be easily uniformed in GUI, we decided not to include the mentioned code in “OpenPhControl”.

Demonstrated concept of using open source tools for reading values from closed source devices by using optical recognition of characters from display, could be very useful in cases of limited financial sources. If there is some old measuring equipment that do not have ability for live communication with PC, or the appropriate software is too expensive, it could be financially justified to make an effort to set system like presented. Desktop devices and required software have a lot functionality that are rarely used, but mobile hand-held devices are modes in this aspect and they are generally cheaper and usable for this purpose. If we are ready to give up comfort offered by modern commercial systems and to invest creative effort, it is possible to get the same functionality with less money. By sharing experience and code with open source community the progress will be faster which will cause greater comfort in work.

The additional hardware extensibility of “OpenPhControl” system could be made by adding an additional sensor. For example, if there is a need for temperature monitoring or control, the inexpensive One Wire Digital Temperature Sensor—DS18B20 (less than 10 EUR with waterproof protection) could be used for temperature measurements. It provides measurement of temperatures from -55°C to +125°C with accuracy of ±0.5°C [45].

Some tests were made so that “OpenPhControl” could work as a PID regulator, and this was done by defining adequate coefficients (Kp, Ki, Kd, time slice). The negative feedback loop of PID regulation of the system was not able to provide stability of the first phase of reaction, primarily due to a slow response of the pH meter and a need to quickly add the significant amount of NaOH. Although it was practical for us not to use the PID regulation in some other situations, here this concept could not be neglected.

The current version of “OpenPhControl” adds a reagent in constant-volume steps which can be manually changed. One of the improvements can be to add the reagent in variable volume steps. The volume steps could vary as a function of the slope of the curve and the optimal volume for dosing could be determined from the measured value changes of the previous dosings.

Other possible further developments include the following: (1) the automatization of tasks such as refilling of the Syringe pump module and its work in tandem [16] (2) the determination of equivalence point based on the titration curve by using various methods [46, 47].

The “OpenPhControl” joins the growing ranks of inexpensive, freely-modifiable open-source analytical instruments that both complement and compete with commercial instruments [48]. All in all, by providing all source code and designs under an open-source license, together with an expandable online repository, we aim to provide a flexible, modular platform upon which enthusiastic colleagues may build and exchange modifications in time. We will be pleased to add modifications to our basic design to the online project repositories as appropriate. For this, please contact the authors directly.

## Conclusion

Reliable and inexpensive open-source pH stat, designed from readily available laboratory devices (a pH meter, a computer, a webcam), open-source “OpenPhControl” software, inexpensive hardware (a peristaltic pump, Arduino, a step motor…) and some 3D printed parts were constructed and evaluated.

The open-source software “OpenPhControl”, controlled by Arduino microcontrollers with a USB connection, was designed to use pump modules. Two pump modules were designed (a low cost peristaltic pump and an upgraded open-source syringe pump) by defining their hardware and specialized firmware, which enabled them to be used. Considerations were made for ease of assembly and reduced costs without compromising performance.

In addition to the basic functions of pH stat, i.e. the pH value measurement and maintenance, an improvement is achieved in terms of using the open-source pH stat for potentiometric titration.

The demonstration of open-source pH stat utility for TEMPO-mediated oxidation of cellulose fibers shows that the operator’s constant attention over the entire experiment and the operator’s subjective error are avoided, while the excellent experimental repeatability can be achieved by using our open-source pH stat.

All in all, we believe that our open-source pH stat will be useful in scientific teaching and research, as well as for professionals working in low-resource environment, because the device allows, at a small fraction of the cost of available commercial offers, significant improvements to be made in experimental work in which the use of pH stat is necessary. The low cost (less than 150 EUR) and modular nature of “OpenPhControl” and its modules, with existing laboratory equipment, is a good reason to support or take over a wide range of additional functions in the laboratory (bill of materials—Support information S1 Table). The most basic functionality for maintaining the pH constant is achievable with peristaltic module (which costs ~15 EUR). Considering that the open-source scientific tools are available to everyone, and that researchers can construct and adjust the device according to their needs, we hope that by time, further improvements and new designs of open-source pH stat will emerge from the global open hardware community.

## Supporting information

S1 Fig Scheme for wiring peristaltic pump module. (TIF)

S1 File Graphical User Interface (GUI). (ZIP)

S1 Table Bill of materials. (XLSX)
