# Supplementary figures and images for: "Do-It-Yourself" reliable pH-stat device by using open-source software, inexpensive hardware and available laboratory equipment
Source: PLoS One. 2018 Mar 6;13(3):e0193744. doi: 10.1371/journal.pone.0193744 (PMC5839570; doi:10.1371/journal.pone.0193744)

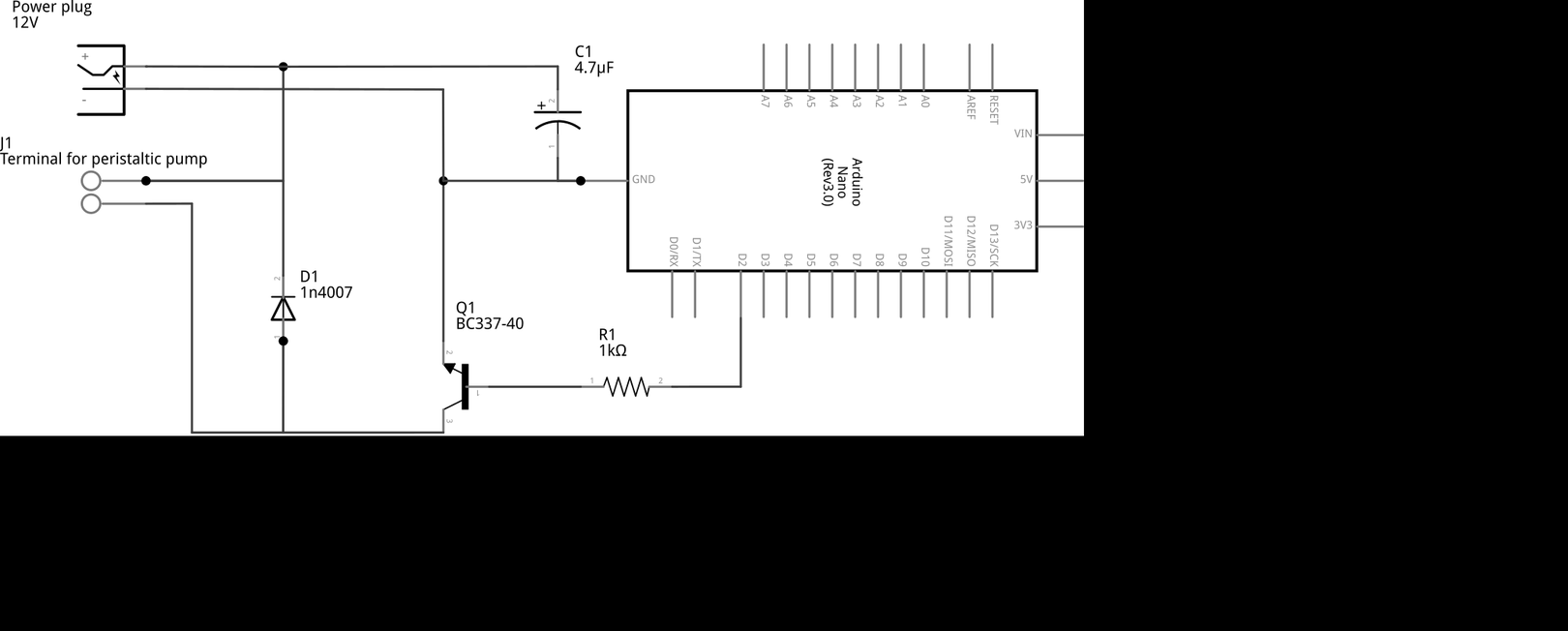

Supplement: S1 Fig — (TIF) [file pone.0193744.s001.tif]

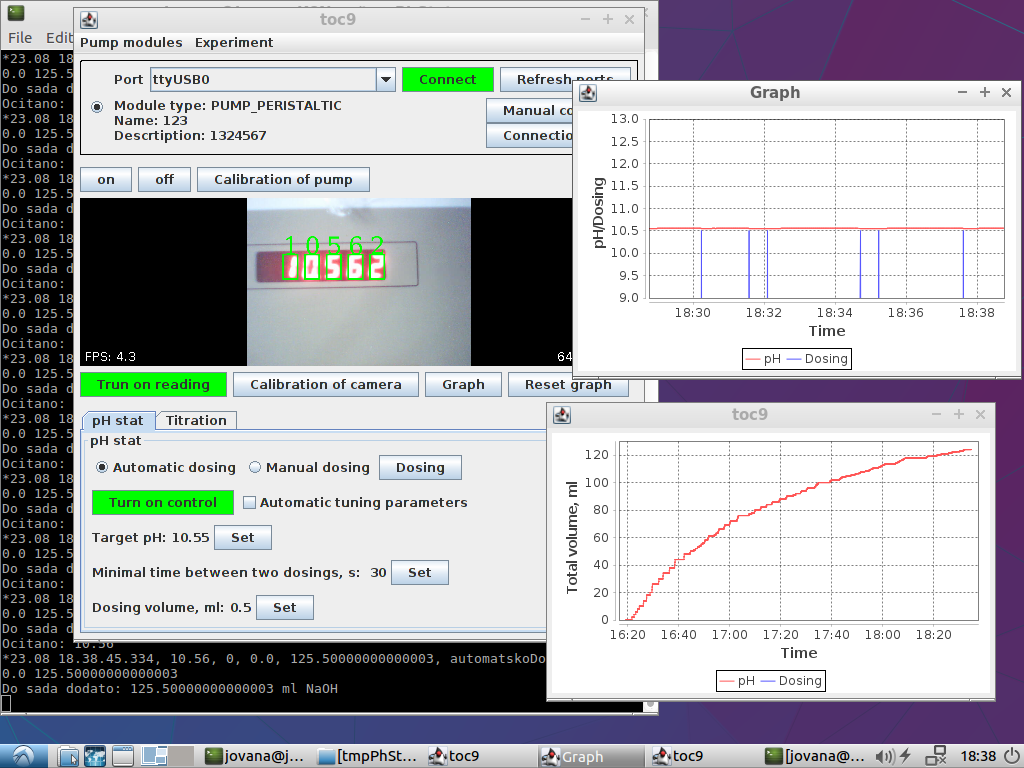

Supplement: S1 File — (ZIP) [file pone.0193744.s002.zip › Fig A The main window during work.png]

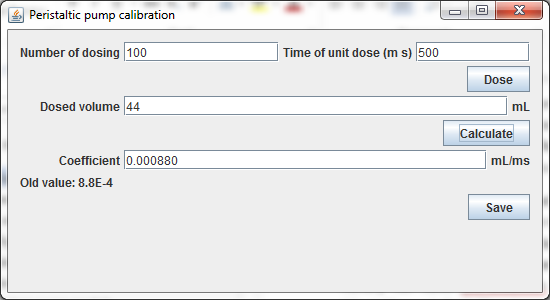

Supplement: S1 File — (ZIP) [file pone.0193744.s002.zip › Fig J Peristaltic pump calibration.png]

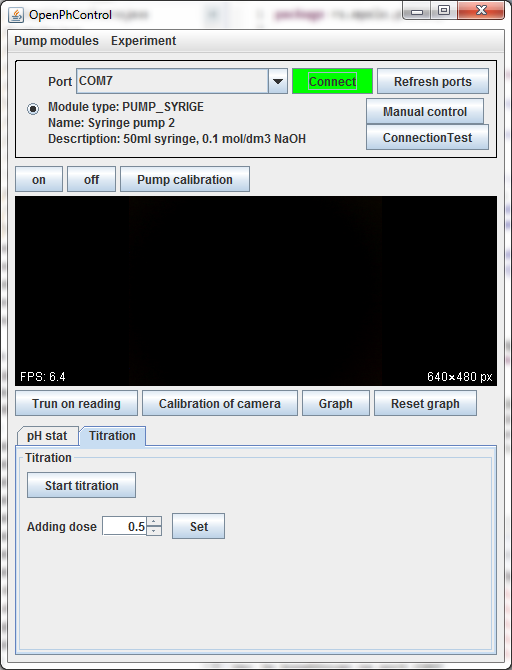

Supplement: S1 File — (ZIP) [file pone.0193744.s002.zip › Fig B The main window for titration.png]

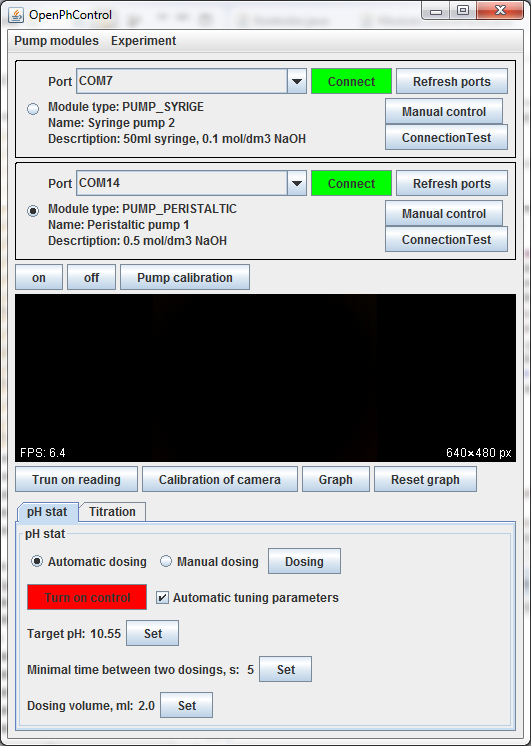

Supplement: S1 File — (ZIP) [file pone.0193744.s002.zip › Fig C The main window with two connected pumps.png]

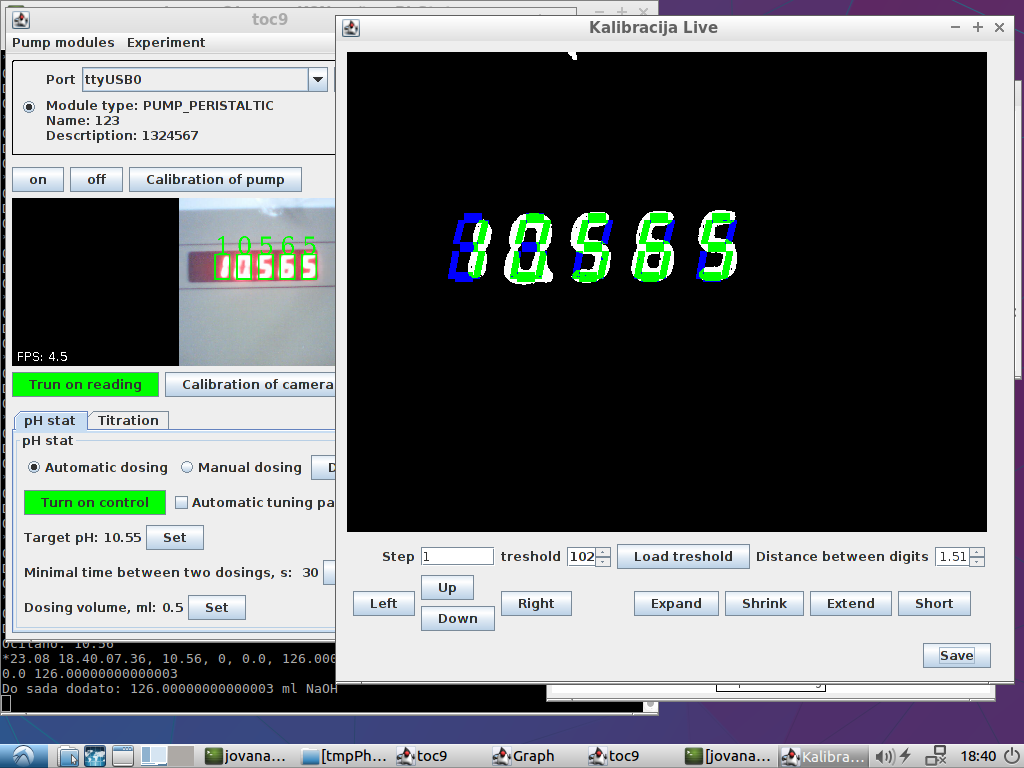

Supplement: S1 File — (ZIP) [file pone.0193744.s002.zip › Fig D Calibration of readings.png]

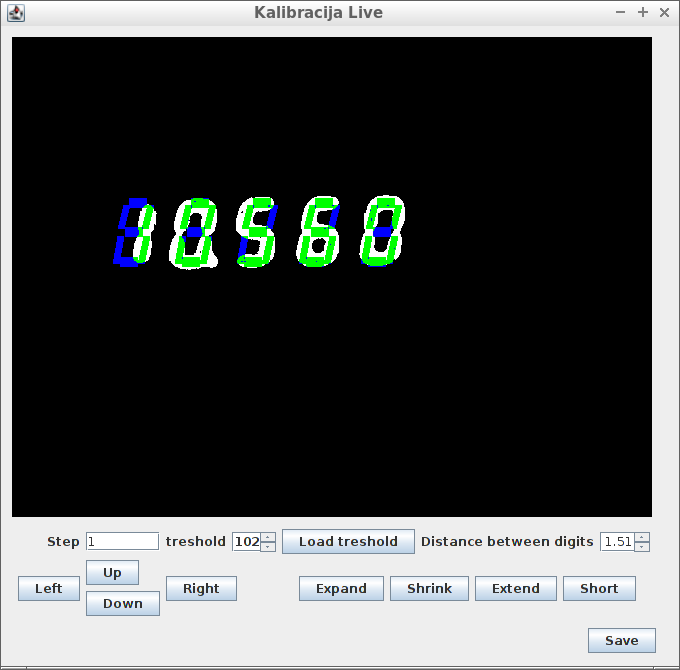

Supplement: S1 File — (ZIP) [file pone.0193744.s002.zip › Fig E The window for calibration of readings.png]

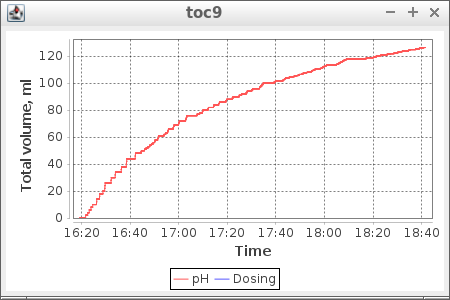

Supplement: S1 File — (ZIP) [file pone.0193744.s002.zip › Fig F The graph of added volume.png]

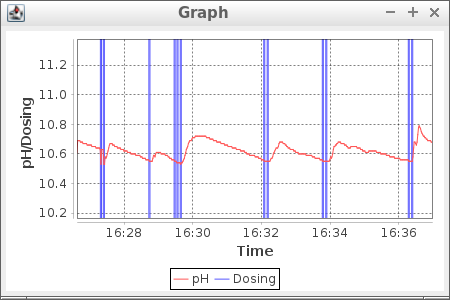

Supplement: S1 File — (ZIP) [file pone.0193744.s002.zip › Fig G The zoomed graph which demonstrates the change of pH.png]

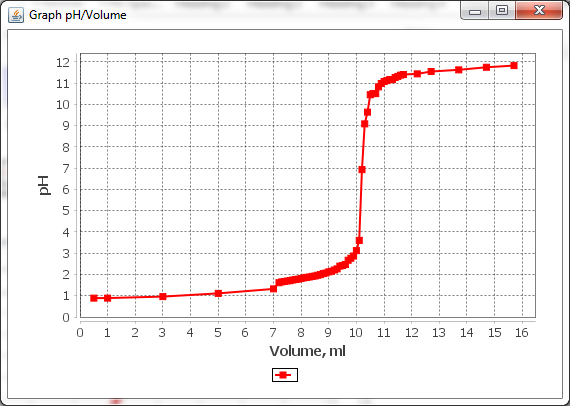

Supplement: S1 File — (ZIP) [file pone.0193744.s002.zip › Fig H The graph with a live titration curve.png]

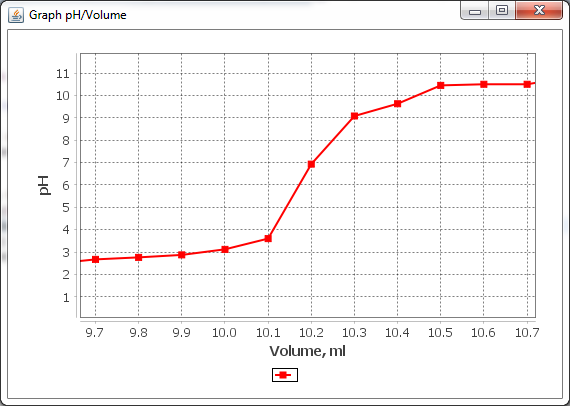

Supplement: S1 File — (ZIP) [file pone.0193744.s002.zip › Fig I The zoomed graph with a live titration curve.png]
